# Solvothermal Preparation of ZnO Nanorods as Anode Material for Improved Cycle Life Zn/AgO Batteries

**DOI:** 10.1371/journal.pone.0075999

**Published:** 2013-10-17

**Authors:** Shafiq Ullah, Fiaz Ahmed, Amin Badshah, Ataf Ali Altaf, Ramsha Raza, Bhajan Lal, Rizwan Hussain

**Affiliations:** 1 Department of Chemistry, Quaid-i-Azam University, Islamabad, Pakistan; 2 National Engineering and Scientific Commission, Islamabad, Pakistan; 3 Department of Chemistry, Bahauddin Zakariya University Sahiwal Campus, Sahiwal, Pakistan; Brandeis University, United States of America

## Abstract

Nano materials with high surface area increase the kinetics and extent of the redox reactions, thus resulting in high power and energy densities. In this study high surface area zinc oxide nanorods have been synthesized by surfactant free ethylene glycol assisted solvothermal method. The nanorods thus prepared have diameters in the submicron range (300∼500 nm) with high aspect ratio. They have uniform geometry and well aligned direction. These nanorods are characterized by XRD, SEM, Specific Surface Area Analysis, solubility in alkaline medium, EDX analysis and galvanostatic charge/discharge studies in Zn/AgO batteries. The prepared zinc oxide nanorods have low solubility in alkaline medium with higher structural stability, which imparts the improved cycle life stability to Zn/AgO cells.

## Introduction

Nanoscale materials are studied extensively for high performance batteries and fuel cells [1]–[3]. Performance of electrochemical devices largely depends upon the surface area of the active materials. Decreasing particle size of the battery grade active materials results in a substantial increase in the surface area and porosity, which speeds up the reaction kinetics by providing high surface area and shorter distance for ionic species during charge/discharge process [4]. Zinc oxide is a unique material with important scientific and technological applications in a variety of fields like photocatalysis, photovoltaic, photodegradation, fluorescence, gas sensing and electronics [5]–[9]. Nano sized ZnO has a variety of applications in electrical, optical, magnetic and chemical fields [9]–[10]. The reduction in particle size of ZnO modifies its physical and chemical properties due to surface area enhancement and quantum confinement [10]–[11]. Portable power technology has not kept up with the demands of the modern portable electronic devices. Therefore, there is a need to develop an advanced battery system for portable devices like laptops and notebooks. Despite the development of advanced battery systems such as lithium ion and metal hydride, zinc silver oxide is still the best with respect to power density. Zn/AgO batteries have a long successful history of use for the military, space programs and underwater marine applications [12]–[16]. The batteries with zinc anode are among the best choices for applications where volume and weight are critical and high power output is required, such as missiles, space and underwater applications. Favorable characteristics of silver zinc batteries are very high specific energy (up to 300 Wh/kg) and volumetric energy density (up to 750 Wh/dm), low self-discharge rate (∼5% per month) and flat voltage during most of the discharge. The main problems of zinc based secondary batteries are shape change, dendritic growth and high solubility of the oxidation products of zinc in the electrolyte that result in shape change and dendritic growth of anode. These problems results in the limited cycle life of the zinc based rechargeable batteries which is the major hurdle in the commercialization of the silver zinc technology for the portable and mobile applications. These shortcomings are due to deficiencies of two of the cell's major components: the zinc electrode and the separators. The zinc electrode bears the major responsibility for the capacity degradation of the silver–zinc cells with cycling. The reasons are shape change or redistribution of the zinc and formation of dendrites. Shape change is a phenomenon by which zinc oxide, formed during the discharge, is partially dissolved in the electrolyte and re-deposited during the recharge in a different location from where it originated. The result is a gradual depletion of negative active material at the top and sides of the electrode, with a corresponding reduction in cell capacity. Methods of reducing shape change have been only marginally successful. These include use of excess zinc, use of oversized negative electrodes, the introduction of special additives and binders into the mass of the electrode, use of low concentration KOH or electrolyte additives that lower the concentration of OH^−^ ions and use of special charging methods [17]–[20]. Special additives include the addition of oxides like HgO, Sb_2_O_3_, TiO_2_, Pb_3_O_4_, neodymium/lanthanum conversion coatings and Y(OH)_3_ [21]–[24]. The electrolyte was modified with various additives like ZnO, LiOH, surfactants, gelating agents, phosphates and sodium tetraborate [18].

ZnO nanorods have been utilized in Li-ion rechargeable batteries as anode material due to high surface area [25]–[27]. Solubility of the ZnO in KOH is one of the major reasons for the shape change of zinc electrodes. One dimensional nanostructures with uniform shape and surface have lower solubility as compared to irregular shaped particles. The main reason for the lower solubility is a decrease in the edges and corners of the zinc oxide particles which are the point of dissolution. In this study we have solvothermaly prepared ZnO nanorods with decreased solubility and enhanced structural stability which results in tremendous improvement in the shape change and cycle life of zinc anode batteries.

## Results and Discussion

### X-Ray diffraction

**Figure 1:**
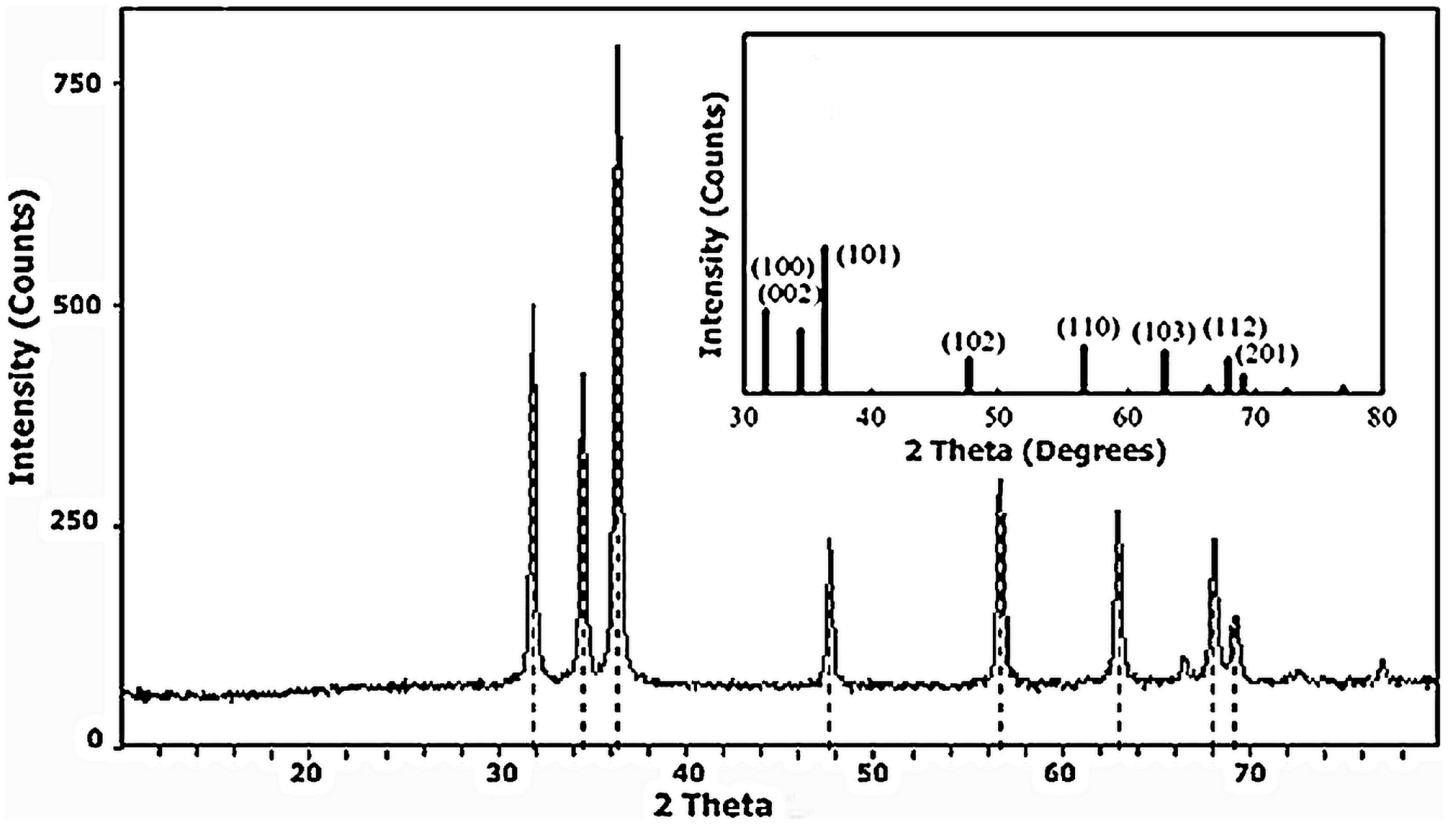
XRD patterns of ZnO nanorods, Inside ZnO (PDF 36–1451).

Structural characterization of the prepared powder was performed by XRD measurement. The spectra were recorded on X-PERT PRO diffractometer using the Cu-Kα radiation. Well defined diffraction peaks observed in Fig. 1 indicates the crystalline nature of the material. All the diffraction peaks correspond to the hexagonal wurtzite structure of ZnO (PDF 36–1451). This is a hexagonal lattice, belonging to the P63mc space group. The broad nature of diffraction peaks is an indication of the nanocrystalline nature of ZnO. The characteristic hexagonal zinc oxide peaks (100), (002), (101), (102), (110), (103), (112) and (201) are present in the X-ray diffractogram with no extra peaks ruling out the possibility of impurities. MDI JADE software was used for the retrieval of XRD data. The crystallite sizes calculated from the Scherer formula and unit cell parameter are summarized in Table 1. The presence of nanocrystallites increases the surface area and thus producing improved current density.

**Table 1:** XRD Data of ZnO nanorods.

2-θ	d(Å)	Miller indices			FWHM	Crystallite size (Å)	Cell Volume (Å^3^)	Density (g/cc)	Unit cell data
		h	k	l					
31.83	2.810	1	0	0	0.327	265	47.42	2.849	a = b = 3.24 c = 5.198 α = β = 90° γ = 120
34.50	2.599	0	0	2	0.272	329			
36.33	2.472	1	0	1	0.333	263			
47.63	1.908	1	0	2	0.341	266			
56.68	1.622	1	1	0	0.400	233			
62.95	1.475	1	0	3	0.402	239			
68.03	1.376	1	1	2	0.359	278			
69.19	1.356	2	0	1	0.405	246			

Crystallite size of the ZnO nanorods was calculated by Scherer formula as below;
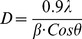
Where D = Crystallite size in Å, λ = 1.54 Å for CuKα, and β = FWHM (Line width at half maximum).

### Scanning Electron Microscopy

**Figure 2:**
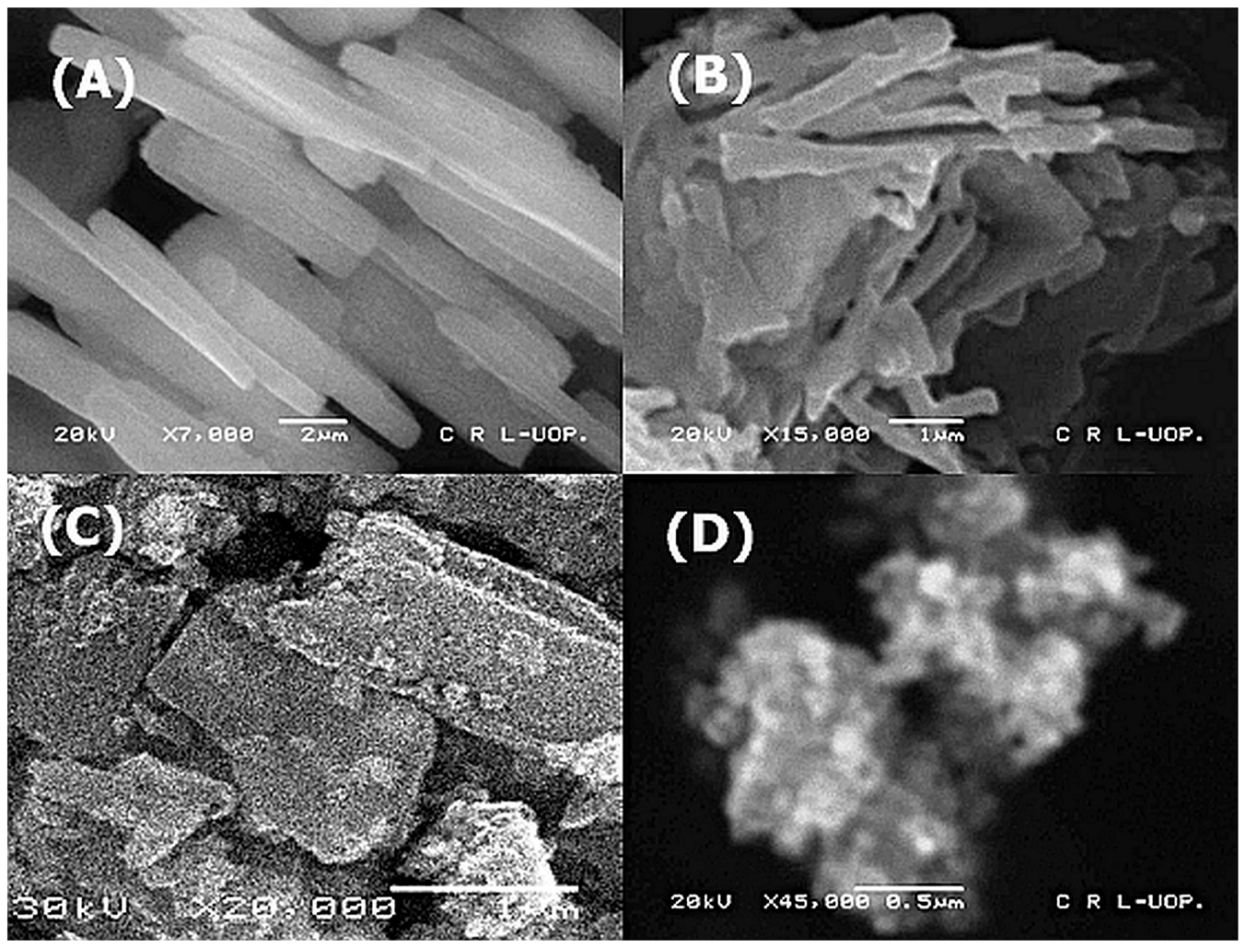
SEM micrographs. (A). ZnO nanorods (B). ZnO nano rods after 18 cycles (C). ZnO micro particles (D). ZnO microparticles after 15 Cycles.

Morphology of the ZnO nanorods was studied with the help of Scanning Electron Microscopy. The ZnO nanorods have a uniform morphology with aligned direction (Fig. 2A). The size of the nanorods is below 500 nm with aspect ratio greater than 20. The advantage of high aspect ratio is decrease in the corners of the materials which are the point of dissolution. Thus, ZnO nanorods with high aspect ratio have lower solubility and better structural stability. These nanorods have smooth and defect less surface. Although, after charging/discharging process the size and shape of these nanorods have been changed but they still retain their structural integrity and there is no growth of needle like dendrites which are responsible for rupturing of the separator and short circuiting of the silver zinc cells (Fig. 2B). This improved behavior results in prevention of shape change of the electrode and thus results in enhanced cycle life stability.

Fig. 2C shows the commercial ZnO micropowder with particle size of 2 to 3 microns with irregular shape. Particles are rough with numerous edges and corners, which results in the enhanced dissolution of the ZnO powder in alkaline medium. Fig. 2D shows the ZnO commercial powder after 15 Cycles. The particles are reduced in size and porous in nature as the dissolved ZnO has been re-deposited at other places of electrodes. This result in poor mechanical strength of the electrode and powder sheds during the charge/discharge process which results in capacity loss and ultimate failure of the cell. The crystallite size calculated from Scherer formula is in close agreement with the particle size calculated with SEM for the ZnO nanorods sample (Table 2). This is indicative of the absence of the grain boundaries within the individual nanorods, which are the points for the dissolution. On the other hand, ZnO micro powder has crystallite size well below the particle size, which suggests the presence of a number of grains inside single particles, thus resulting in the enhanced dissolution of the ZnO particles. The ZnO micro powder also contains a number of small irregular shaped particles attached to the larger particles. These particles easily shed during the charge/discharge process resulting in capacity loss.

**Table 2:** Physical characteristics of the zinc oxide nanorods.

Sample	Crystallite size (XRD) (nm)	Particle size (SEM) (nm)
ZnO powder	450	2200
ZnO nanorods	264	450

### Solubility of ZnO 1D nanostructures in alkaline medium

**Figure 3:**
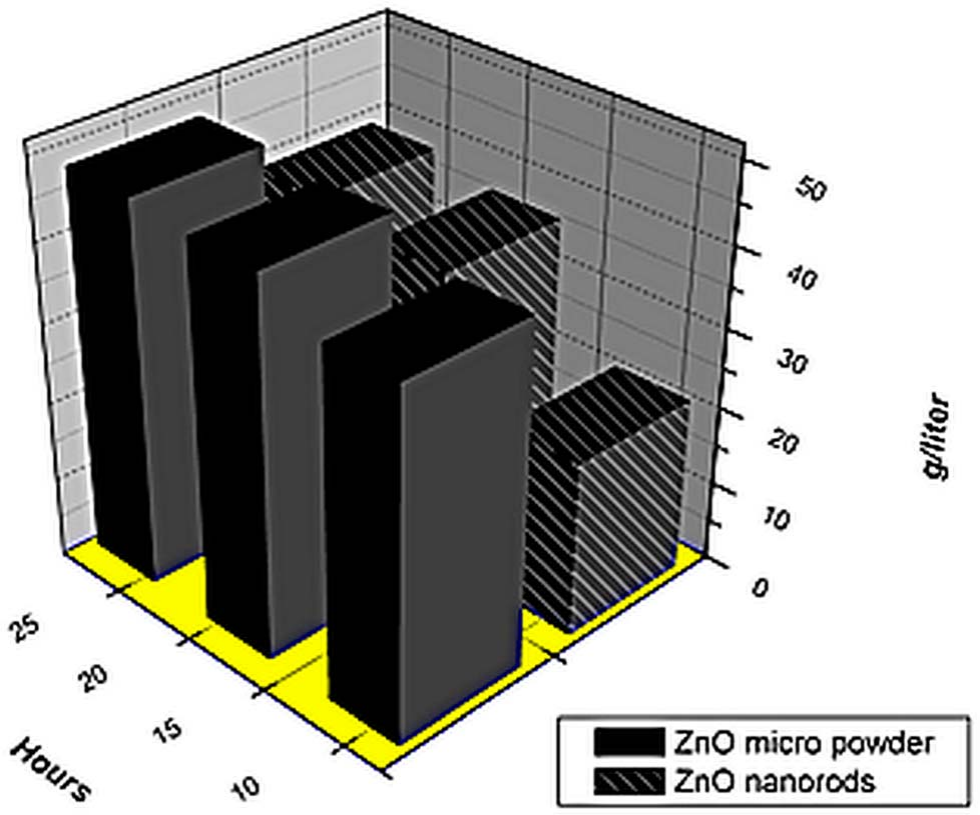
Comparison of solubility of ZnO micro powder and nanorods in alkaline medium.

Solubility of ZnO nanorods was evaluated in 6M KOH solution. 10 grams ZnO was added to 100 ml KOH solution in a sealed container and stirred for three intervals of 8 hours at 45°C. The residual ZnO was separated by centrifugation. It was observed that ZnO nanorods due to uniform morphology and smooth surface dissolved gradually and to a lesser extent (Fig. 3). This resistance to dissolution results in the structural stability of the nanorods and prevents the shape change of zinc electrode. It can be observed from Fig. 3 that ZnO micropowder due to rough surface and presence of loosely attached small particles dissolve at a higher rate and up to a larger extent in the KOH solution of similar strength.

### Specific Surface area analysis

The specific surface area was measured on surface area analyzer Autosorb-1 Model No. AS-10-LP. The Sample was degassed at 150°C for 2 hours under nitrogen atmosphere. BET surface area, Langmuir surface area and micropore volume of the ZnO samples is summarized in Table 3. It can be clearly seen that ZnO nanorods due to smaller particle size have higher surface area and pore volume. Synthesized nanorods have four times higher BET surface area as compared to a commercial ZnO powder which results in improved discharge capabilities.

**Table 3:** Specific surface area of ZnO nanorods and micropowder.

Sample	BET Area (m^2^/g)	Langmuir Area (m^2^/g)	Micro Pore Volume (mm^3^/g)
ZnO nanorods	18.42	24.84	1.66
ZnO micropowder	4.21	5.42	0.11

### Elemental analysis

**Figure 4:**
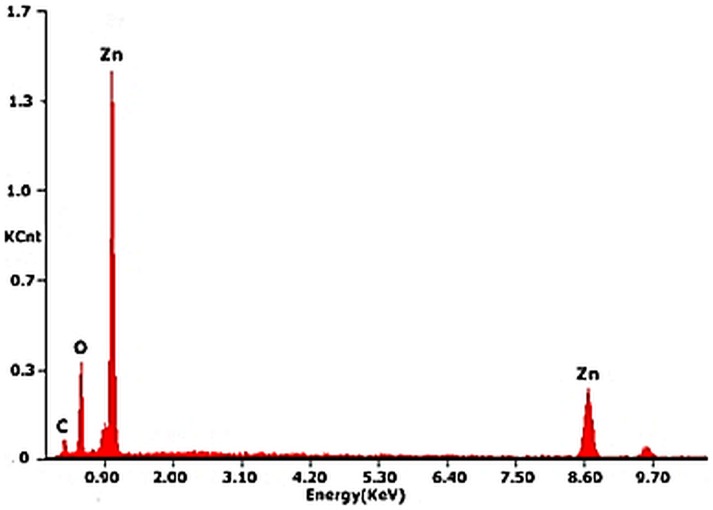
EDX pattern of ZnO nanorods.

Elemental composition of the synthesized ZnO nanorods was carried out with EDX analysis (Table 4). The EDX spectrum (Figure 4) has characteristic peaks of Zn and oxygen. EDX results are in close agreement with the literature data [28]–[29]. The experimental results clearly confirm the formation of pure ZnO with Zn to oxygen ratio in close agreement with theoretical values.

**Table 4:** Elemental analysis of the synthesized nanorods.

Element	Theoretical Composition	EDX composition
Zinc	80.34	80.32
Oxygen	19.66	19.68
Total	100	100

### Electrochemical testing

As synthesized ZnO nanorods were used as anode material for rechargeable silver zinc batteries. These batteries have a poor cycle life because of zinc electrode shape change and cell short circuiting occurs as the zinc dendritic needles grow during the charge/discharge process on anode. ZnO powder is soluble in alkaline electrolyte and is dissolved during the discharge process. It is re-deposited on the zinc electrode edges during charging which results in shape change. These thickened edges are accompanied by sharp needle like zinc dendrites during repeated charge discharge cycles. These needles like dendrites penetrate the separator and contact with the silver cathode results in short circuiting and early failure of the silver zinc cell. This phenomenon is also observed in other rechargeable zinc anode batteries like Zn-MnO_2_ and Ni-Zn etc. Due to greater stability and less solubility of the 1D nanostructures ZnO nanorods were investigated for the cycle life enhancement in silver zinc batteries. The ZnO nanorods show better performance and cycle life as compared to commercial zinc oxide powder.

**Figure 5:**
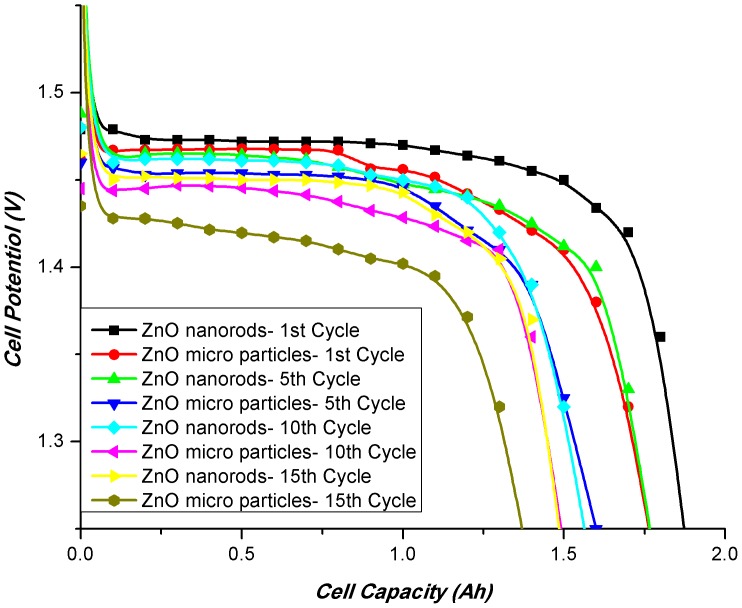
Discharge of the silver zinc cells at 1C discharge rate.

**Figure 6:**
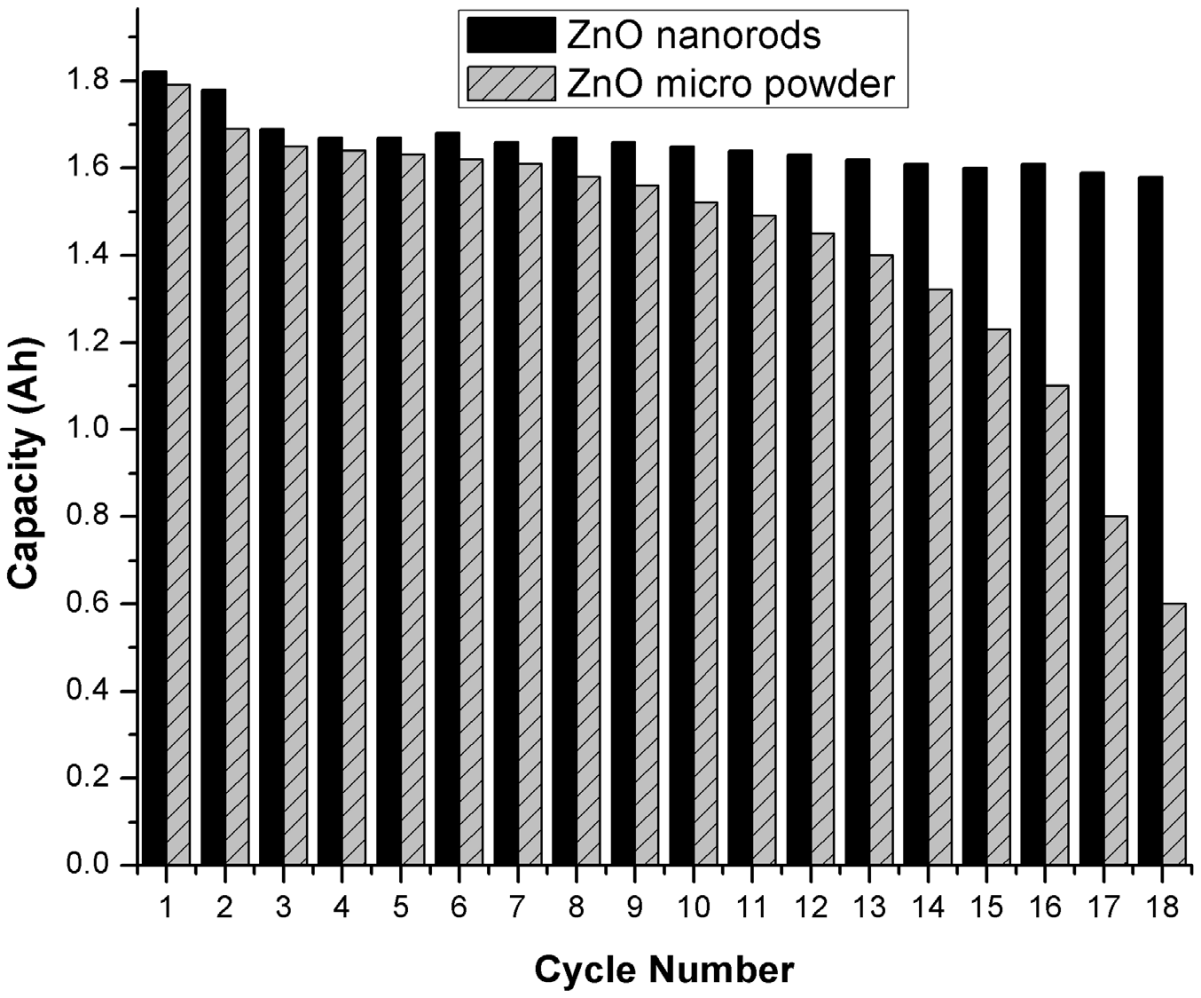
Cycle life comparison of ZnO nanorods vs ZnO micro powder.

Prismatic zinc silver cell with 1.6Ah nominal capacity was used to investigate the electrochemical performance of the synthesized zinc oxide nanorods. The cell was charged at 0.1C and discharged at 1C for 18 cycles. For comparison cell was also fabricated with the conventional zinc oxide powder with mean particle size of 2 to 3 micrometers. The charge/discharge behavior of the zinc oxide powder and zinc oxide nanorods is shown in Fig. 5. It is evident from the results that cell fabricated with zinc oxide nanorods shows a better open circuit and onload voltages. Zinc oxide nanorods gave higher first cycle capacity than zinc oxide micro powder and retain its stability up to 18 cycles. Cell fabricated with zinc oxide powder shows poor cycle life stability and its capacity is reduced to less than 80% after 15^th^ cycle. In comparison the cell fabricated with zinc oxide nanorods shows greater cycle life stability and gave more than 90% capacity in the 18th cycle (Figure 6). The reason for the enhanced stability of the zinc oxide nanorods is the reduced solubility of the 1D ZnO nanorods and enhanced structural stability. The zinc oxide nanorods retain their morphology after 18th cycles as evident from the scanning electron microscopy (Figure 2B). This reduced solubility and enhanced structural integrity results in prevention of shape change and formation of zinc needles even after 18 cycles which in turns prevents the short circuit of the cell and imparts cycle life stability and enhanced capacity retention.

Fig. 6 shows the comparison of cycle life stability of the zinc oxide micro powder and zinc oxide nanorods. It is clearly evident from the results that cell fabricated with powder zinc oxide is stable up to 6^th^ cycle but after that it degrades ad eventually fails in the 14^th^ cycle. This is the result of the gradual shape change of the electrodes and needle like dendrites formation in the powder zinc oxide cells. In comparison the cell fabricated with zinc oxide nanorods shows higher discharge capacity than the powder zinc cell and retains its capacity for more than 18 cycles due to enhanced stability of the zinc oxide nanorods electrodes. This results in tremendous improvement in cycle life and battery failure.

## Materials and Methods

ZnO nanorods were prepared by solvothermal method using Teflon lined stainless steel autoclave. 0.1 M solution of zinc acetate was prepared in a mixture of ethanol and ethylene glycol (2∶1) at 100°C. The reactor was filled to 80% of the volume and was heated to 170°C for 4 hours during which the pressure developed was 40 psi. At this high pressure and temperature ethylene glycol acts as soft template for the growth of ZnO nanorods. The reaction mixture was cooled to room temperature, centrifuged and washed with de-ionized water. The resultant ZnO powder was annealed at 300°C to remove any residual organic impurities.

Structural characterization of the prepared ZnO nanorods was performed by XRD measurement. The spectra were recorded on Philips X-Pert PRO 3040/60 diffractometer using the Cu-Kα radiation at 40 kV and 40 mA in the 2θ range 10–80° at a scan speed of 60 s/step. Scanning electron microscopy was performed on JEOL JSM-5910 operating at 20 keV for morphology and particle size. The sample was dispersed in ethanol by sonication and a drop was placed on carbon tape. EDX analysis was performed on JEOL JSM-5910 for elemental composition. Solubility of the ZnO nanorods was evaluated in alkali for dissolution studies. BET surface area was analyzed by nitrogen adsorption-desorption isothermal measurements.

ZnO nanorods were analyzed as active anode material for silver zinc rechargeable batteries. ZnO nanorods, electrolytic zinc powder, polyvinyl alcohol binder and mercury oxide (77∶15∶5∶3) were mixed and pressed on silver wire current collecting grid at 20 MPa pressure. The powder was wrapped in cellulose paper before pressing. The pressed electrode was dried in vacuum oven at 80°C for 4 hours. Most of the electrolyte and electrode additives like Bi_2_O_3_, SnO_2_, Pb_3_O_4_, K_2_BO_3_ etc. were avoided to specifically study the effect of the ZnO shape and particle size on the performance of the zinc anode batteries. The prepared electrode was electroformed against sintered silver electrode in 6M KOH solution. Silver electrode capacity was kept at 300% of the anode to make the cell anode limited. These electrodes were washed with de-ionized water till neutral in charged form. Silver electrodes were air dried while zinc electrodes were dried in vacuum oven at 80°C for 4 hours. The electroformed electrodes were wrapped in cellulose triacetate and assembled in nylon casing. The nominal capacity of the cell was 1.6Ah. Electrolyte was ZnO saturated 6M KOH solution. Cells were assembled with flooded electrolyte. Assembled cells were charged at 0.1C and discharged at 1C to check the specific energy density and the cycle life stability of the synthesized ZnO nanorods.

## Conclusion

ZnO nanorods with uniform geometry and aligned direction have been successfully synthesized by ethylene glycol assisted solvothermal method. These nanorods have crystallite size in nano range with high BET surface area. The particle size of the nanorods studied by scanning electron microscopy is in sub-micron range with high aspect ratio. Due to smooth surface and few corners and edges solubility of the nanorods in alkaline medium is decreased which results in improved structural integrity of the material during charge/discharge cycling. The prepared material was investigated as anode material in silver zinc batteries with the aim to reduce the shape change of the electrodes. Electron microscopy and galvanostatic charge/discharge studies clearly indicates that the nanorods have retained their shape and performance during 18 cycles. This improved mechanical behavior resulted in improved cycle life and higher energy and power densities of the silver zinc batteries.
